# Effects of high-flow nasal cannula oxygen therapy in bronchiectasis and hypercapnia: a retrospective observational study

**DOI:** 10.1186/s12890-024-03037-2

**Published:** 2024-05-02

**Authors:** Jing Yang, Lei Chen, Hang Yu, Jingjing Hu, Feng Qiu

**Affiliations:** Department of Respiratory and Critical Care Medicine, The First Affiliated Hospital of Ningbo University, Liuting Street NO.59, Ningbo, 315010 Zhejiang China

**Keywords:** Bronchiectasis, Respiratory failure, High-flow nasal cannula, Noninvasive ventilation

## Abstract

**Background:**

The effectiveness of high-flow nasal cannula (HFNC) therapy in patients with bronchiectasis experiencing hypercapnia remains unclear. Our aim was to retrospectively analyze the short-term outcomes of HFNC therapy in such patients, and to further explore the predictors of HFNC treatment failure in this particular patient population.

**Methods:**

A retrospective review was conducted on patients with bronchiectasis who received HFNC (*n* = 70) for hypercapnia (arterial partial pressure of carbon dioxide, PaCO_2_ ≥ 45 mmHg) between September 2019 and September 2023.

**Results:**

In the study population, 30% of patients presented with acidemia (arterial pH < 7.35) at baseline. Within 24 h of HFNC treatment, there was a significant reduction in PaCO_2_ levels by a mean of 4.0 ± 12.7 mmHg (95% CI -7.0 to -1.0 mmHg). Concurrently, arterial pH showed a statistically significant increase with a mean change of 0.03 ± 0.06 (95% CI 0.01 to 0.04). The overall hospital mortality rate in our study was 17.5%. The median length of hospital stay was 11.0 days (interquartile range [IQR] 8.0 to 16.0 days). Sub-analysis revealed no statistically significant differences in hospital mortality (19.0% vs. 20.4%, *p* = 0.896), length of hospital stay (median 14.0 days [IQR 9.0 to 18.0 days] vs. 10.0 days [IQR 7.0 to 16.0 days], *p* = 0.117) and duration of HFNC application (median 5.0 days [IQR 2.0 to 8.5 days] vs. 6.0 days [IQR 4.9 to 9.5 days], *p* = 0.076) between the acidemia group and the non-acidemia group (arterial pH ≥ 7.35). However, more patients in the non-acidemia group had do-not-intubate orders. The overall treatment failure rate for HFNC was 28.6%. Logistic regression analysis identified the APACHE II score (OR 1.24 per point) as the independent predictor of HFNC failure.

**Conclusions:**

In patients with bronchiectasis and hypercapnia, HFNC as an initial respiratory support can effectively reduce PaCO_2_ level within 24 h of treatment. A high APACHE II score has emerged as a prognostic indicator for HFNC treatment failure. These observations highlight randomized controlled trials to meticulously evaluate the efficacy of HFNC in this specific population.

## Background

Non-cystic fibrosis bronchiectasis, characterized by cough, expectoration, and abnormal thickening and dilation of bronchial walls visible on pulmonary imaging, represents a clinical syndrome with a complex and diverse etiology. Over the past two decades, a notable increase has been observed in the incidence and prevalence of this condition [1]. Acute respiratory failure (ARF) is a common cause of death in patients with bronchiectasis [2]. However, limited data is available regarding the clinical outcomes for bronchiectasis patients with ARF. Very few retrospective studies have focused on this patient population in the intensive care unit (ICU), reporting mortality rates ranging from 19 to 34% [3–5]. Notably, in these studies, a considerable proportion of patients (93 of 140 patients) exhibited hypercapnia and received noninvasive ventilation (NIV) and invasive mechanical ventilation (IMV) therapy.

NIV is currently recommended for the treatment of patients with hypercapnic respiratory failure [6, 7]. However, several factors such as ventilation interface discomfort, excessive airway secretions, and disease severity can contribute to NIV treatment failure [8]. High-flow nasal cannula (HFNC) has emerged as a promising alternative [9], offering both clinical and physiological benefits including alveolar recruitment, reduction of dead space, facilitation carbon dioxide removal [10], increased secretion clearance [11], and patient comfort [12]. Most research on hypercapnic ARF has been conducted in patients with acute exacerbation of chronic obstructive pulmonary disease (AECOPD). Recent studies suggest that HFNC is non-inferior to NIV in terms of carbon dioxide elimination and prevention of endotracheal intubation in AECOPD with mild to moderate respiratory acidosis [13–15]. However, the role of HFNC in the management of patients with bronchiectasis and hypercapnia remains unclear.

Therefore, the objective of this study is to conduct a retrospective analysis of the short-term efficacy of HFNC in reducing hypercapnia and improving acidemia in bronchiectasis patients, and to identify the factors that predict treatment failure with HFNC in this particular patient population.

## Methods

### Study design

This single-center retrospective study was approved by the Ethics Committee of First Affiliated Hospital of Ningbo University, Ningbo, China, with a waiver of informed consent. Our respiratory department has a dedicated unit for patients with critical respiratory conditions. Those who require intensive monitoring or noninvasive respiratory support, but have not yet reached the indications for endotracheal intubation, are admitted to this unit for further management. Using admission and discharge records, we included all consecutive adult patients (aged over 18 years) with bronchiectasis who received HFNC as initial noninvasive respiratory support for hypercapnia in our department from September 2019 to September 2023.

### Patient population and diagnosis

We defined bronchiectasis based on : (1) the presence of symptoms such as chronic sputum production, cough, or hemoptysis, and (2) specific computed tomography (CT) findings: a bronchial inner diameter to concomitant pulmonary artery outer diameter ratio > 1, or visible small bronchi within 1 cm from the costal side of the visceral pleura or adjoining mediastinal pleura [16]. High-resolution CT (HRCT) was prioritized over conventional CT scans. All the images were evaluated by a radiologist.

We defined hypercapnia as an arterial partial pressure of carbon dioxide (PaCO_2_) ≥ 45.0 mmHg, with or without acidemia. Acidemia is defined as an arterial pH < 7.35, based on the arterial blood gas measurement obtained at baseline before the initiation of HFNC therapy. Non-acidemia is defined as arterial pH ≥ 7.35 under the same baseline conditions. Chronic hypercapnia is defined as a sustained elevation of PaCO_2_ above 45.0 mmHg, with a minimum interval of six weeks between two measurements, assessed as a baseline characteristic before the initiation of HFNC therapy.

Exclusion criteria included: (1) initial treatment with NIV; (2) use of HFNC for less than 1 h; (3) post-extubation application; (4) concomitant COPD; (5) incomplete medical records.

Considering the bronchiectasis - chronic obstructive pulmonary disease overlap (BCO), two respiratory physicians independently reviewed patients with a confirmed diagnosis of COPD. They assessed clinical features, smoking history, and CT images. BCO was defined as the concurrent presence of respiratory symptoms, a smoking history of > 10 pack-years, a forced expiratory volume in one second/ forced vital capacity (FEV_1_/FVC) ratio of < 0.7, and confirmation of bronchiectasis via HRCT scan, without an identifiable cause. To minimize the confounding effect of COPD, only those BCO patients exhibiting diffuse bronchiectasis affecting multiple bilateral lung lobes (number of affected lobes > 3) on CT scans were included in the study.

### Treatment regimes

For patients with bronchiectasis and hypercapnia our department has a common initial support protocol. Specific therapies include nebulized salbutamol and ipratropium bromide, systemic steroids, empirical antibiotic therapy targeting Pseudomonas aeruginosa and airway clearance treatment by physiotherapists for respiratory secretions. Patients who failed such conservative measures and had persistent hypercapnic ARF were put on non-invasive respiratory support or IMV.

Non-invasive respiratory support, including NIV and HFNC, was typically initiated in patients with at least one of the following clinical indicators: worsening dyspnea, respiratory rate > 25 breaths per minute, use of accessory respiratory muscles, cyanosis, or altered mental status. The physician’s decision to use HFNC was based primarily on a thorough assessment of the patient’s clinical status and personal preferences, particularly when NIV was contraindicated or not tolerated by the patient due to discomfort or other factors. The HFNC (AIRVO™2, Fisher and Paykel Healthcare, Auckland, New Zealand) was initially set at a flow rate of 45 L/min and a temperature of 37 °C. Adjustments to the flow rate and/or temperature were made based on the patient’s tolerance and response. Generally, HFNC was applied continuously both day and night as long as tolerated by the patient. In the event of any interruptions to either therapy, nasal cannula oxygen therapy was provided to maintain pulse oxygen saturation (SpO_2_) levels within the range of 88–92%.

For patients who exhibited no improvement or worsening of clinical signs of respiratory failure, or deterioration in gas exchange (i.e., an increase in PaCO_2_ by 20% of the baseline) after initial HFNC therapy, NIV was considered as a pre-intubation rescue option. Routine blood gas analysis was performed after 2 h of NIV rescue treatment. If the blood gas measurements indicated further deterioration with clinical signs of worsening, such as changes in consciousness, endotracheal intubation was considered.

Endotracheal intubation and IMV were recommend if the patient exhibited any of the following criteria: worsening PaCO_2_ accompanied by a decrease in arterial pH; arterial oxygen saturation (SaO_2_) < 88% despite an optimal fraction of inspired oxygen (FiO_2_); signs of agitation or deteriorating consciousness; severe hemodynamic instability; severe cardiac arrhythmias; or respiratory or cardiac arrest.

### Data collection

Clinical data were extracted from electronic medical records, which included demographic information, relevant comorbidities, bronchiectasis etiology and chest imaging.

The causes of hospitalization were categorized as follows: acute exacerbation of bronchiectasis without identified precipitating factors [16], pneumonia (new-onset pulmonary infiltrative lesions with fever, leukocytosis, or leukopenia) [17], heart failure (pulmonary edema or peripheral edema, and echocardiographic abnormalities) [18], hemoptysis, pulmonary embolism and sedative overdose.

Physiological data including arterial blood gas measurements and vital signs at baseline (prior to the initiation of HFNC therapy), were collected. Furthermore, the initial settings of HFNC, any ventilatory support changes, and arterial blood gas measurements within 24 h of initial HFNC therapy were also recorded. To maintain analytical consistency, the last arterial blood gas measurement collected within the first 24 h was included in the analysis. For patients who received HFNC therapy for less than 24 h, their final arterial blood gas measurement before HFNC discontinuation was included.

### Study outcomes

The primary outcomes in this retrospective analysis were changes in PaCO_2_ and arterial pH within the first 24 h. Secondary outcomes included treatment failure rate, in-hospital death, duration of HFNC application, and length of hospital stay. Additionally, the study aimed to identify predictors of HFNC treatment failure. Treatment failure was defined as the requirement for intubation, death with do-not-intubate orders, or change from HFNC to NIV.

### Statistical analysis

Continuous variables with a normal distribution were presented as mean ± standard deviation (SD). Non-normal variables were reported as the median (interquartile range). Comparisons between different groups were performed using chi-square test, Fisher’s exact test, t test, paired t test and Mann-Whitney U test, depending on the nature of the data. To identify predictors of in-hospital mortality, univariate analyses were first conducted on related variables, comparing HFNC success and HFNC failure. Variables with *p* values less than 0.15 were then entered into a forward stepwise logistic regression analysis with an entry level of 0.05 and a removal level of 0.10. A *p*-value of less than 0.05 was considered statistically significant. Statistical analyses were performed using SPSS version 26.0.

## Results

### Patient characteristics

**Fig. 1:**
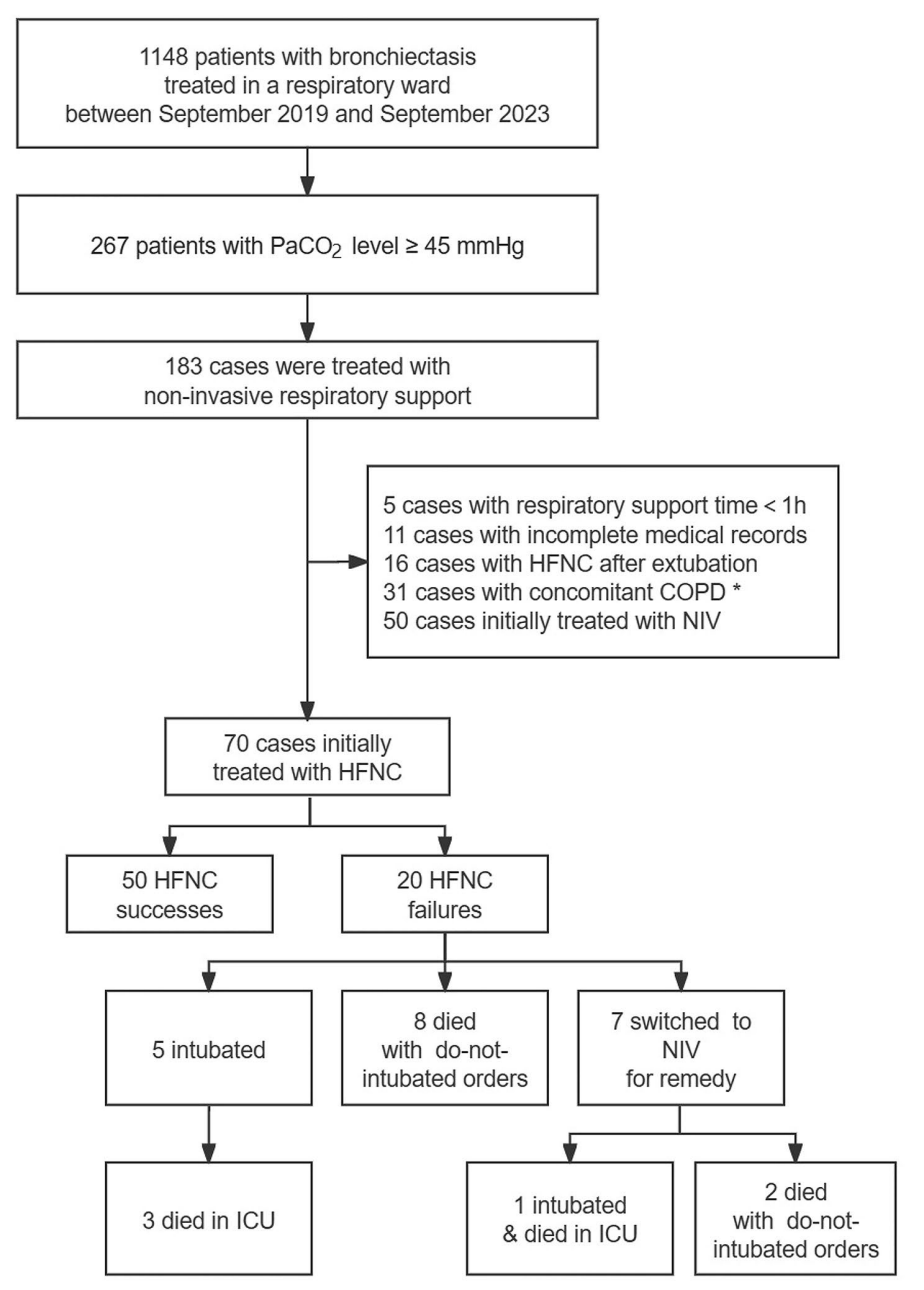
Inclusion and outcomes of patients. HFNC high-flow nasal cannula; NIV noninvasive ventilation; COPD chronic obstructive pulmonary disease. * Only patients with bronchiectasis-chronic obstructive pulmonary disease overlap who exhibit diffuse bronchiectasis affecting multiple bilateral lung lobes on CT scans were included in the study

A total of 70 patients with bronchiectasis and hypercapnia received HFNC as the initial noninvasive respiratory support therapy (Fig. 1). Almost half of the patients were female (47.1%), with a mean age of 69.2 years. The predominant etiologies of bronchiectasis in this study were post-tuberculous disease (28 patients, 40.0%), idiopathic (21 patients, 30.0%), and previous infection (14 patients, 20.0%). The CT manifestations included varicose (32 patients), columnar (21 patients), and cystic (17 patients), with a median of 4 affected lobes. Pulmonary function reports were available for 17 patients. These reports revealed low spirometry values, with the median predicted FVC at 39.7% and the median predicted FEV_1_ at 28.3% [19]. Among these patients, more than half had chronic hypercapnia (44 patients, 62.9%). Fourteen patients were on long-term oxygen therapy, and one was on domiciliary NIV. Pneumonia was considered the major cause for hospitalization in this study. Pseudomonas aeruginosa and Aspergillus were the most common pathogens detected. At baseline, the mean arterial pH and PaCO_2_ were 7.37 ± 0.07 and 73.3 ± 13.8 mmHg, respectively. Acidemia was observed in 30% of the included patients. Table 1 displays the baseline characteristics of the study subjects grouped according to acidemia and non-acidemia.

**Table 1:** Baseline characteristics of patients with bronchiectasis and hypercapnia treated with high-flow nasal cannula therapy

	Acidemia ^a^ (*n* = 21)	Non-acidemia (*n* = 49)	*p* value
Demographics			
Age, years	68.3 ± 10.7	69.6 ± 14.7	0.724
Female, n (%)	8 (38.1)	26 (53.1)	0.251
Body mass index, kg/m^2^	20.1 ± 3.6	19.5 ± 3.8	0.560
Ex-Smoker, n (%)	7 (33.3)	7 (14.3)	0.102
Asthma, n (%)	3 (14.3)	4 (8.2)	0.421
BCO, n (%) ^b^	1 (4.7)	5 (10.2)	0.661
Chronic hypercapnia, n (%) ^c^	11 (52.4)	33 (67.3)	0.235
Long-term oxygen therapy, n (%)	2 (9.5)	19 (38.8)	0.317
Domiciliary NIV, n (%)	0	1 (2.0)	1.000
Use of immunosuppressive agents, n (%) ^d^	3 (14.3)	4 (8.2)	0.421
Etiology, n (%)			
Post-tuberculous	11 (52.4)	17 (34.7)	0.166
Idiopathic	3 (14.3)	18 (36.7)	0.060
Previous infection	7 (33.3)	7 (14.3)	0.102
CTD	0	2 (4.1)	1.000
DPB	0	3 (6.1)	0.549
PCD	0	2 (4.1)	1.000
Pulmonary function, n ^e^	7	10	
FEV_1_% predicted	29.5 (24.8, 40.6)	26.9 (22.5, 39.2)	0.454
FVC% predicted	40.8 (37.3, 49.7)	39.7 (35.2, 57.0)	1.000
FEV_1_/FVC	50.1 (47.0, 81.5)	57.6 (50.0, 65.9)	0.635
Cause of hospitalization, n (%)			
Pneumonia	13 (61.9)	22 (44.9)	0.192
Heart failure	3 (14.3)	12 (24.5)	0.201
Acute exacerbation	3 (14.3)	12 (24.5)	0.527
Hemoptysis	2 (9.5)	1 (2.0)	0.212
Pulmonary embolism ^f^	0	2 (4.1)	1.000
Vital signs			
Temperature, °C	37.0 ± 0.5	37.0 ± 0.4	0.571
Heart rate, per min	97.5 ± 15.5	98.2 ± 15.8	0.949
Respiratory rate, per min	20.9 ± 4.9	22.2 ± 3.9	0.058
Mean blood pressure, mmHg	84.8 ± 12.7	90.8 ± 13.5	0.087
Laboratory measurements			
White blood cell	8.8 ± 4.6	9.1 ± 4.3	0.509
C-reactive protein	24.8 (11.1, 63.7)	21.0 (11.8,46.4)	0.939
Neutrophil-to-lymphocyte ratio	9.5 ± 7.7	13.5 ± 22.6	0.613
Arterial blood gas measurements			
pH	7.30 (7.26, 7.33)	7.40 (7.37, 7.42)	0.000
PaCO_2_, mmHg	78.0 ± 15.3	76.1 ± 12.1	0.583
HCO_3_-, mEq/L	31.6 ± 5.0	36.1 ± 4.7	0.001
Lactate, mmol/L	1.3 ± 0.6	1.4 ± 1.0	0.847
PaO_2_/FiO_2_, mmHg	212.2 ± 75.4	248.7 ± 76.7	0.069
APACHE II score	11.0 (8.0, 11.5)	10.0 (8.0, 15.5)	0.933

NIV, Noninvasive ventilation; BCO, Bronchiectasis-chronic obstructive pulmonary disease overlap; FEV_1_, Forced expiratory volume in one second; FVC, Forced vital capacity; CTD, Connective tissue disease; DPB, Diffuse panbronchiolitis; PCD, Primary ciliary dyskinesia; CVID, Common variable immunodeficiency; APACHE, Acute Physiology and Chronic Health Evaluation;

^a^ Acidemia is defined as arterial pH < 7.35 at baseline; non- acidemia is defined as arterial pH ≥ 7.35 at baseline

^b^ Diagnosis of BCO is based on clinical manifestations, smoking history, CT findings, and lung function of the patients

^c^ Chronic hypercapnia is defined as a sustained elevation of PaCO_2_ above 45.0 mmHg, with a minimum interval of six weeks between two measurements

^d^ Use of immunosuppressive agents is defined as treatment with steroids, immunosuppressive medications, and/or chemotherapeutic agents within six months

^e^ Spirometric parameters were assessed based on the GLI using the Quanjer 2012 dataset

^f^ Two patients were admitted with a diagnosis of pulmonary embolism, both with a history of chronic pulmonary heart disease. They presented with dyspnea and severe heart failure during the hospitalization

### Ventilatory support settings and effects

The initial median gas flow rate was 40.0 L/min (interquartile range [IQR] 35.0 to 45.0 L/min) for HFNC therapy. Within the first four hours of HFNC therapy, two patients received endotracheal intubation due to worsening dyspnea. The remaining sixty-eight participants continued to receive HFNC treatment for over 24 h. The median duration of HFNC therapy at the time of post arterial blood gas sample analyzed was 21.0 h (IQR 18.4 to 24.0 h).

**Fig. 2:**
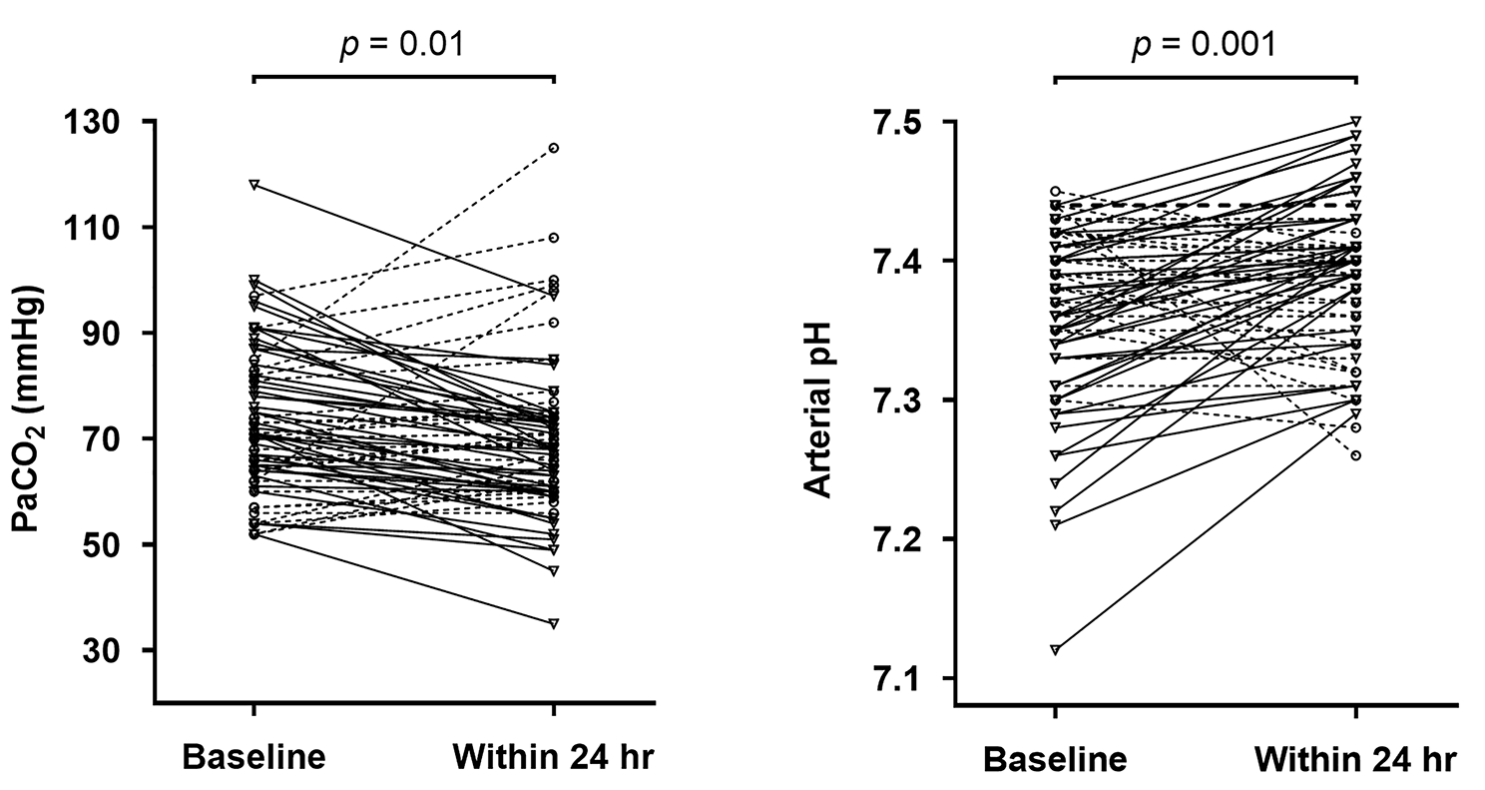
Changes in arterial blood gas measurements at baseline and within 24 h of high-flow nasal cannula treatment. Bold lines represent individual cases whose blood gas parameters improved within 24 h of treatment. Dotted lines represent individual cases that showed no improvement in blood gas parameters after 24 h of treatment

Figure 2 displays the effects of HFNC on the blood gas measurements. Within 24 h treatment, PaCO_2_ demonstrated a statistically significant decrease (mean change − 4.0 ± 12.7mmHg; 95% CI -7.0 to -1.0 mmHg), while arterial pH demonstrated a statistically significant increase (mean change + 0.03 ± 0.06; 95% CI 0.01 to 0.04).

### Clinical outcomes

The overall treatment failure rate for HFNC therapy was 28.6% (20 patients). Table 2 presents the details of treatment failures. Among these, seven patients were switched to NIV as rescue therapy, with four successfully avoided the need for tracheal intubation and ultimately survived. The mean time from the initiation of HFNC to switch to NIV was 3.3 ± 1.7 days. Five patients underwent tracheal intubation directly due to worsening respiratory failure, with two intubated within the first 24 h of HFNC therapy. Subgroup analysis for acidemia stratification showed no statistically significant difference in treatment failure rate between the two subgroups (38.1% vs. 24.5%, *p* = 0.248). However, within the acidemia subgroup, the need for intubation was higher (19.0% vs. 2.0%, *p* = 0.026).

**Table 2:** Outcomes of study patients on high-flow nasal cannula therapy grouped by acidemia and non-acidemia

Outcome	Acidemia ^a^ (*n* = 21)	Non-acidemia (*n* = 49)	*p* value
Treatment failure, n (%)	8 (38.1)	12 (24.5)	0.248
Do-not-intubate orders	0	8 (18.4)	0.200
Invasive ventilation	4 (19.0))	1 (2.0)	0.026
Treatment switch ^b^	4 (19.0)	3 (6.1)	0.186
Hospital mortality, n (%) ^c^	4 (19.0)	10 (20.4)	0.896
Duration of HFNC application, days	5.0 (2.0, 8.5)	6.0 (4.5, 9.5)	0.076
Length of hospital stay, days	14.0 (9.0–18.0)	10.0 (7.0–16.0)	0.117

HFNC, high-flow nasal cannula;

^a^ Acidemia is defined as arterial pH < 7.35 at baseline; non- acidemia is defined as arterial pH ≥ 7.35 at baseline

^b^ Treatment switch is defined as a change from HFNC to NIV.

^c^ Hospital mortality includes patients with do-not-intubate orders

The overall in-hospital mortality rate for the inclusion patients was 20.0% (14 patients). Specifically, within the patients experiencing HFNC treatment failure, eight patients died with do-not-intubate orders, two patients died following rescue NIV therapy, and four died after endotracheal intubation and mechanical ventilation, amounting to a mortality rate of 70% in this subgroup. The primary causes of mortality were identified as: cardiac arrest associated with respiratory failure (7 patients), multiple organ failure (including respiratory) (5 patients), fatal hemoptysis (1 patient), and cerebrovascular accident (1 patient).

The median duration of HFNC application was 6.0 days (IQR 3.8 to 9.0 days), and the median length of hospital stay was 11.0 days (IQR 8.0 to 16.0 days). Sub - analysis revealed no statistically significant differences in hospital mortality, duration of HFNC application, or length of hospital stay between the two subgroups (Table 2).

On univariate analysis, patients with higher APACHE II scores and neutrophil to lymphocyte ratios (NLR) were more likely to experience HFNC failure. Logistic regression analysis showed that the APACHE II score was an independent predictor of HFNC failure (Table 3).

**Table 3:** Variables associated with failure of high-flow nasal cannula therapy

Characteristic	Univariate analysis				Multivariate analysis	
	Success (*n* = 50)	Failure (*n* = 20)	*p* value		OR (95% CI)	*p* value
Use of immunosuppressive agents, n (%)	3 (6.0)	4 (20.0)	0.097		-	0.063
Fungal infections, n (%)	1 (2.0)	3 (15.0)	0.067		-	0.106
APACHE II score	9.7 ± 2.9	12.6 ± 5.1	0.008		1.24 (1.06–1.46) per point	0.003
NLR	9.8 ± 13.6	19.8 ± 29.2	0.034		-	0.146

APACHE, Acute Physiology and Chronic Health Evaluation; NLR, Neutrophil to lymphocyte ratio

## Discussion

Our study results indicate that HFNC therapy effectively reduced PaCO_2_ and increased arterial pH levels within 24 h in patients with bronchiectasis and hypercapnia. The APACHE II score was identified as an independent predictor of treatment failure for HFNC therapy in this patient population.

NIV is considered the standard noninvasive ventilation support for managing patients with acute hypercapnic respiratory failure [6]. However, its application may be limited by factors such as reduced comfort and suboptimal patient-ventilator interaction [20]. Furthermore, the necessity for airway clearance in patients with bronchiectasis can pose additional challenges when using an oronasal mask, which is a common interface for NIV. HFNC is a noninvasive, high-concentration oxygen delivery interface that can provide up to 50–60 L/min of airflow and reliably achieve up to 100% FiO_2_. HFNC has shown several valuable effects in patients with AECOPD [21], and has also been studied in the long-term treatment of bronchiectasis [22, 23]. Claudia Crimi et al. reported, in an observational study, significant improvements in gas exchange and dyspnea scores in patients with AECOPD and documented bronchiectasis using HFNC [11]. However, their study did not include acidotic patients. To our knowledge, no previous data exists that describe the feasibility of HFNC in treating bronchiectasis with hypercapnia. In our study, the frequency use of HFNC for patients with hypercapnia was likely due to our staff’s expertise with this therapy and the predominance of mild hypercapnia (arterial pH ≥ 7.35 and PaCO_2_ ≥ 45 mmHg) in the majority (70%) of the study population. Our findings indicate that HFNC may be an effective initial respiratory support for bronchiectasis patients with mild hypercapnia, reducing PaCO_2_ and improving arterial pH levels within 24 h of treatment.

In our study, the overall failure rate of HFNC therapy was 28.6%. Patients in the acidemia group more frequently underwent intubation, however, this trend was counterbalanced by a higher prevalence of do-not-intubate orders in the non-acidemia group. As a result, no statistically significant difference was observed in the treatment failure rates between the two subgroups. Notably, in the acidemia subgroup, 19.0% of patients received NIV as rescue therapy, which is lower than the rate reported in a randomized controlled trial comparing HFNC and NIV in moderate AECOPD (arterial pH 7.25–7.35, PaCO_2_ ≥ 55 mmHg). In that trial, 32.5% of patients were switched to NIV due to worsening respiratory failure within 6 h of HFNC therapy, and it was hypothesized that this might be related to the low arterial pH and insufficient effectiveness of HFNC. In contrast, our inclusion population comprised a higher proportion of patients with chronic hypercapnia, who typically exhibit greater tolerance to acidosis and hypercapnia, potentially influencing the response to noninvasive respiratory therapy. Additionally, pneumonia was quite common in our inclusion population. Given the high secretory nature of bronchiectasis, pulmonary infection induced secretions can lead to airway obstruction and ventilatory disorder. In this case, adequate secretion clearance is crucial. Although both NIV and HFNC can provide airway humidification, which aids in secretion management. However, the frequent need for expectoration in bronchiectasis patients may lead to interruptions in respiratory support when using NIV oral-nasal masks. In contrast, HFNC allows for continuous secretion expectoration and airway clearance without disrupting respiratory support. Univariate analysis revealed that HFNC failure is more common in patients with higher APACHE II scores and NLR. NLR, an indicator of systemic inflammation and infection, along with the APACHE II score, are considered effective tools in assessing the severity of various diseases, including bronchiectasis [24], AECOPD [25], community acquired pneumonia [26], and sepsis [27]. Multivariate analysis further identified the APACHE II score as an independent predictor of HFNC treatment failure. Therefore, the decision to administer HFNC in patients with bronchiectasis and hypercapnia, especially those with high APACHE II scores, should be made with caution, considering the potential for treatment failure and the need for close monitoring.

The hospital mortality in our study was 17.5%, which is lower than the previously reported rates of 19–34% [3–5]. This discrepancy might be associated with the lower baseline APACHE II scores in our cohort, suggesting less severe conditions among our study patients. The length of hospital stay seemed to be longer in the acidemia group, but did not reach statistical significance. This observation may be attributed not only to the more severe conditions in the acidemia group but also to the higher proportion of pneumonia in this group, which is often associated with a worse prognosis and longer hospital stays compared to acute exacerbations [28]. However, it is important to note that the non-acidemia group included more patients with do-not-intubate orders, which could potentially confound the true mortality rates and hospital stay durations. Future rigorously designed randomized controlled studies are needed to better evaluate the efficacy of HFNC in bronchiectasis with hypercapnia.

There are several limitations to our study. Firstly, due to the retrospective nature of our study, the indications and applications of HFNC therapy were not standardized. Our physicians did prefer HFNC to NIV for patients with bronchiectasis and mild hypercapnia. This clinical preference could introduce a selection bias into our study. Secondly, the retrospective nature of the study resulted in a lack of parameters at certain timepoints, such as one hour after treatment, because arterial blood gasses are performed less frequently in our department. Other important details, including sputum volume, previous exacerbations, previous exposure to HFNC treatment, 28-day and long-term mortality, were also lacking. Additionally, studies reported that a higher HFNC flow rate (60 L/min) could better improve lung aeration, dynamic compliance, and reduce the indexes of respiratory effort [29, 30]. In our study, the median HFNC flow rate was 40 L/min, which may be a potential factor affecting the outcomes. Finally, the small number of cases limits the statistical power and the ability to detect significant differences. Considering various other confounding factors, our multivariate analysis results must be interpreted with caution.

## Conclusions

The results of our study indicate that HFNC, as an initial respiratory support, can effectively help reduce carbon dioxide retention in patients with bronchiectasis and mild hypercapnia. Further large-scale randomized controlled trials are needed to assess the efficacy of HFNC in comparison to NIV in this specific population.

## Data Availability

The datasets used or analyzed during the current study are available from the corresponding author on reasonable request.

## Abbreviations

HFNC: High-flow nasal cannula
NIV: Noninvasive ventilation
APACHE: Acute Physiology and Chronic Health Evaluation
ARF: Acute respiratory failure
ICU: Intensive care unit
IMV: Invasive mechanical ventilation
AECOPD: Acute exacerbation of chronic obstructive pulmonary disease
HRCT: High-resolution computed tomography
BCO: Bronchiectasis - chronic obstructive pulmonary disease overlap
PaCO_2_: Arterial carbon dioxide pressure
SpO_2_: Pulse oxygen saturation
SaO_2_: Arterial oxygen saturation
FiO_2_: Fraction of inspired oxygen
NLR: Neutrophil to lymphocyte ratio
